# Recentes Avanços na Pesquisa Experimental em Cardiologia

**DOI:** 10.36660/abc.20200835

**Published:** 2020-10-13

**Affiliations:** 1 Universidade Estadual Paulista Faculdade de Medicina de Botucatu BotucatuSP Brasil Faculdade de Medicina de Botucatu - Universidade Estadual Paulista (UNESP), Botucatu, SP - Brasil; 2 Harvard Medical School Brigham and Women's Hospital BostonMA EUA Brigham and Women's Hospital - Harvard Medical School, Boston, MA – EUA

**Keywords:** Doenças Cardiovasculares/tendências, Exercício, Insuficiência Cardíaca, Mecanismos Moleculares/tendências, Domínios Científicos, Atividades Científicas e Tecnológicas/tendências

A pesquisa básica é de grande importância para fomentar e ampliar o conhecimento nas mais diversas áreas. A prática da cardiologia clínica modificou-se substancialmente em resposta aos avanços das pesquisas experimentais das últimas décadas, as quais possibilitaram maior entendimento dos mecanismos moleculares envolvidos nas doenças cardiovasculares. Consequentemente, novas ferramentas foram introduzidas para diagnóstico e novos fármacos tiveram suas indicações definidas para o tratamento das doenças cardiovasculares.^1^

A observação dos artigos publicados nos Arquivos Brasileiros de Cardiologia nos últimos anos mostra grande avanço da produção científica na área básica, tanto de artigos originários de investigadores brasileiros como de pesquisadores de várias nações. Adicionalmente, verifica-se a diversidade de origem da produção científica nacional relacionada a campos do conhecimento; na última década, o jornal teve expressivo aumento da quantidade de artigos de áreas como educação física, fisioterapia, nutrição, biologia e biomedicina, dentre outras. Neste editorial, comentaremos sobre estudos recentemente publicados nos Arquivos Brasileiros de Cardiologia na área de pesquisa básica.

A prática de exercícios tem sido considerada cada vez mais importante na prevenção e no tratamento de doenças cardiovasculares, particularmente quando se leva em conta o envelhecimento da população.^2^ Consequentemente, o papel do exercício físico na evolução das doenças cardiovasculares teve destaque na produção científica nacional e internacional nos últimos anos. Artigos recentemente publicados nos ABC mostraram que o exercício físico contribui para o equilíbrio redox e inflamatório no coração em situações de agressão sistêmica, como a obesidade^3^ e a ovariectomia associada a nocaute do receptor da lipoproteína de baixa densidade.^4^ Além disso, foi relatado que, na cardiopatia diabética, o exercício estimula a angiogênese miocárdica.^5^ Mesmo atividades consideradas passivas, como a vibração de corpo inteiro, tiveram efeitos benéficos em ratos, como o aumento da tolerância à isquemia miocárdica.^6^ O conjunto dos resultados contribui para o entendimento dos mecanismos pelos quais o exercício tem efeitos benéficos na prevenção e no tratamento das doenças cardiovasculares.

Terapia nutricional é outra estratégia extensamente abordada na cardiologia.^7^ Um estudo recentemente publicado destacou os efeitos antioxidantes do açaí e a melhora do metabolismo energético no modelo global de isquemia-reperfusão em ratos, que foram independentes de alterações na função ventricular esquerda.^8^ Outro componente avaliado experimentalmente foi a gordura. Muniz et al.^9^ observaram que dieta rica em banha aumenta o peso corporal sem, no entanto, levar a dislipidemia em ratos. Por outro lado, alimentação rica em banha e em colesterol induz dislipidemia e danos graves ao fígado.^9^

Além das potenciais estratégias não farmacológicas descritas, novas indicações de medicamentos foram contempladas nos estudos publicados nos ABC. Ramezani-Aliakbari et al.^10^ avaliaram o uso da trimetazidina em ratos com cardiomiopatia diabética. O fármaco, comumente utilizado para melhorar o metabolismo miocárdico na insuficiência coronariana, reduziu o grau de hipertrofia miocárdica e melhorou parâmetros eletrocardiográficos e de função ventricular. Outro medicamento avaliado foi o losartan, um bloqueador do receptor tipo 1 da angiotensina II, que melhorou a função miocárdica de ratos com obesidade induzida por dieta.^11^

No campo da revascularização miocárdica, foi observado que a administração de rapamicina em combinação com α-cianoacrilato foi superior para manter a patência vascular ao uso isolado de cada um dos fármacos em enxertos vasculares de ratos.^12^ Os efeitos positivos pareceram ligados a diminuição do espessamento intimal, da proliferação celular e da resposta inflamatória no enxerto.^12^

Finalmente, foram publicados estudos relacionados à investigação de fatores agravantes de doenças cardiovasculares. Vassallo et al.^13^ observaram que a exposição ao mercúrio agrava a hipertensão arterial sistêmica e aumenta a atividade plasmática da enzima conversora da angiotensina e o estresse oxidativo miocárdico de ratos espontaneamente hipertensos. Outro fator de risco para doenças cardiovasculares abordado foi o estresse físico,^14^^,^^15^ cuja indução durante o período pré-natal resultou em alterações sexo-específicas na expressão gênica de receptores adrenérgicos β1 da prole adulta de ratos.^14^ Por outro lado, a aplicação de estresse físico 60 minutos antes da indução de isquemia-reperfusão teve efeitos benéficos, como a redução da área do infarto e a melhora da função ventricular em ratos.^15^

Apesar do aumento nas informações sobre vias de sinalização moduladas pelo exercício físico, dos efeitos sistêmicos e cardiovasculares de diferentes terapias nutricionais e dos novos usos de fármacos para prevenção e tratamento de doenças cardiovasculares, ainda há um longo caminho até que o conhecimento possa ser incorporado à prática clínica. Espera-se que o avanço na medicina translacional possa contribuir para a redução do tempo entre o conhecimento básico e sua aplicação clínica. Para isso, os Arquivos Brasileiros de Cardiologia têm importante papel na publicação de artigos com relevância e importância científica em todas as áreas do conhecimento relacionadas à cardiologia. Adicionalmente, o periódico tem promovido a interprofissionalização e o debate científico de qualidade, integrando diferentes profissionais envolvidos na prevenção e nos cuidados de doenças do sistema cardiovascular.
