# Supplementary material for: Recentes Avanços na Pesquisa Experimental em Cardiologia
Source: Arq Bras Cardiol. 2020 Oct 13;115(4):593–4. [Article in Portuguese] doi: 10.36660/abc.20200835 (PMC8386960; doi:10.36660/abc.20200835)
Supplement: Translation

# Translation: Overview of Recent Advances in Experimental Cardiovascular Research

**Affiliations:** 1 BotucatuSP Brazil Faculdade de Medicina de Botucatu - Universidade Estadual Paulista (UNESP), Botucatu, SP - Brazil; 2 BostonMA USA Brigham and Women's Hospital - Harvard Medical School, Boston, MA – USA

**Keywords:** Cardiovascular Diseases/trends, Exercise, Heart Failure, Molecular Mechanisms/trends, Scientific Domaine, Scientific and Technical Activities/trends

Basic research is essential to generate and expand knowledge in several research areas. In recent decades, cardiology practice has substantially changed in response to advances in experimental research, which have provided a better understanding of the molecular mechanisms involved in cardiovascular diseases. Consequently, new diagnostic evaluation tools have been introduced and new drugs have been indicated for treating cardiovascular diseases.^1^

Recent articles in *Arquivos Brasileiros de Cardiologia*, also known as ABC Cardiol, have shown great scientific advances in basic research, with studies originating from researchers from Brazil and from other countries. Additionally, knowledge from more diverse areas has been seen in national scientific output. In the last decade, the journal has seen a significant increase in the number of articles from different fields of research such as physical education, physiotherapy, nutrition, biology, biomedicine, etc. This Editorial focuses on basic research articles recently published in ABC Cardiol.

Currently, exercise is considered an important tool for preventing and treating cardiovascular diseases, particularly when considering population aging.^2^ Consequently, the use of physical exercise has played an important role in cardiovascular diseases in the contemporary national and international scientific output. Recently, ABC Cardiol articles have shown that physical exercise contributes to redox and inflammatory balance in the heart in conditions of systemic agressions, such as obesity^3^ and low-density lipoprotein receptor knockout associated with ovariectomy.^4^ Exercise has also been shown to stimulate myocardial angiogenesis in diabetic cardiomyopathy.^5^ Even passive exercise, such as whole-body vibration has produced beneficial effects by increasing myocardial tolerance to ischemia in rats.^6^ These results add to the understanding of the mechanisms involved regarding the beneficial effects of exercise on cardiovascular disease prevention and treatment.

Another widely addressed strategy in cardiology is nutritional therapy.^7^ A recent study highlighted the antioxidant effects of açaí berry and the improvement in energy metabolism, regardless of changes in left ventricular function after ischemia-reperfusion in rats.^8^ Fat is another component under the experimental spotlight. Muniz et al.^9^ reported that a high-lard diet increases body weight without inducing rat dyslipidemia. On the other hand, a high-lard high-cholesterol diet leads to dyslipidemia and severe liver damage.^9^

In addition to the potential non-pharmacological therapies described above, new drug effects have been studied in reports published in ABC Cardiol. Ramezani-Aliakbari et al.^10^ evaluated the use of trimetazidine in diabetic rats with cardiomyopathy. The drug, often used to improve myocardial metabolism in coronary heart disease, reduced myocardial hypertrophy, and improved electrocardiographic and functional ventricular parameters ^10^. Another drug, the angiotensin-II type 1 receptor blocker losartan, was shown to improve myocardial function in rats with high-fat diet-induced obesity.^11^

In the field of myocardial revascularization, rapamycin administered in combination with α-cyanoacrylate was superior in maintaining vascular patency than either used individually in vascular grafts in rats.^12^ The positive effects seemed to be related to decreased intimal thickening, cell proliferation, and inflammatory response in the graft.^12^

Finally, studies investigating factors aggravating cardiovascular diseases have also been published. Vassallo et al.^13^ described that mercury exposure impairs systemic arterial hypertension and increases myocardial oxidative stress and plasma activity of angiotensin-converting enzyme in spontaneously hypertensive rats. Physical stress, another risk factor for cardiovascular diseases, was also addressed.^14^^,^^15^ Physical stress induction during the prenatal period resulted in sex-specific changes in the β1 adrenergic receptor gene expression of adult rat offspring.^14^ On the other hand, applying physical stress 60 minutes before ischemia-reperfusion reduced the infarction area and improved ventricular function in rats.^15^

Despite the increase in information on the effect of physical exercise on signaling pathways, the systemic and cardiovascular effects of different diets, and the use of novel drugs for preventing and treating cardiovascular diseases, there is still a long way to go before knowledge can be incorporated into clinical practice. Hopefully, advances in translational medicine can help reduce the time lag between basic knowledge and its clinical application. ABC Cardiol has an important role in publishing scientifically relevant and important articles related to all areas of cardiology. Additionally, the journal promotes high-quality multi-professional and scientific debate, bringing together different professionals involved in the prevention and treatment of cardiovascular diseases.
